# Volumetric modulated arc therapy (VMAT) for hippocampal-avoidance whole brain radiation therapy: planning comparison with Dual-arc and Split-arc partial-field techniques

**DOI:** 10.1186/s13014-020-01488-5

**Published:** 2020-02-18

**Authors:** Adams Hei Long Yuen, Po Man Wu, Alex Kai Leung Li, Philip Chung Yin Mak

**Affiliations:** 1 Department of Diagnostic Radiology and Clinical Oncology, Li Ka Shing Faculty of Medicine, The University of Hong Kong, Pokfulam, Hong Kong Special Administrative Region, China; 2 Oncology Centre, St. Teresa’s Hospital, 327 Prince Edward Road, Hong Kong Special Administrative Region, China

**Keywords:** Hippocampal sparing, Partial-field, Split-arc, Volumetric modulated arc therapy, Whole brain radiation therapy, Neurocognitive deficit

## Abstract

**Background:**

Although whole brain radiation therapy (WBRT) provides palliation and prophylaxis, reduces local recurrence probability and improves overall survival, it is evident that WBRT is associated with neurocognitive deficits due to radiation induced damage of the hippocampus. Therefore, minimizing hippocampal dose to the least possible level is of high clinical relevance. In dual-arc conventional volumetric modulated arc therapy (dac-VMAT), the large irradiation field for whole brain planned target volume (PTV) requires a wide jaw opening in which substantial low dose volume to the hippocampus may be produced due to suboptimal multi-leaf collimator (MLC) movements. The present study investigates the potential of a radiation therapy technique with split-arc and reduced field size, namely split-arc partial-field volumetric modulated arc therapy (sapf-VMAT) to spare the hippocampus during WBRT.

**Methods:**

Computed tomography and magnetic resonance images of 20 patients with brain metastases were retrieved in this retrospective planning study. The hippocampus was manually delineated by single radiation oncologist strictly following the RTOG 0933 atlas definition. Plans delivering 30 Gy in 10 fractions were generated for each patient using dac-VMAT and sapf-VMAT. Dosimetric parameters from both techniques were compared by paired t-test.

**Results:**

The results demonstrated that radiation dose to the hippocampus was significantly reduced using sapf-VMAT relative to dac-VMAT plans. sapf-VMAT (7.86Gy, *p* = 0.001) had significantly lowered average D_100%_ compared to dac-VMAT (9.23 Gy). Decrease in hippocampus D_max_ using sapf-VMAT (13.23 Gy, *p* = 0.001) was statistically significant when compared to dac-VMAT (16.33 Gy). The resulting mean dose to the hippocampus was 9.16 Gy for the for sapf-VMAT. Mean dose of sapf-VMAT was significantly lower than dac-VMAT (10.85 Gy, *p* < 0.05). In both eyes, sapf-VMAT demonstrated significantly lower D_mean_ compared to dac-VMAT (*p* < 0.05). Whole brain PTV coverage was not compromised in both techniques.

**Conclusion:**

sapf-VMAT has demonstrated significant dose reduction to the hippocampus and both eyes compared to dac-VMAT.

## Introduction

Although whole brain radiation therapy (WBRT) provides palliation and prophylaxis, reduces local recurrence probability and improves overall survival [1–5], it is evident that WBRT is associated with neurocognitive deficits [6–10] due to radiation induced damage of neural stem cell (NSC) compartment in the hippocampus [11–14]. It is hypothesized that the NSCs in the hippocampus are exquisitely radiosensitive, radiation inflammation causes alteration of the microenvironment and subsequently forces premature differentiation of neuronal progenitor cells and adoption of glial fates [15]. Previous published clinical study of Gondi et al. [16] has demonstrated that dose to 100% volume (D_100%_) of the hippocampus exceeds 9 Gy and maximum dose (D_max_) of the hippocampus exceeds 16 Gy in WBRT treatment course of 30 Gy in 10 fractions were associated with impair memory function. In addition, accumulated preclinical and clinical data have also suggested that neurocognitive deficits manifests at much lower radiation doses than previously expected (less than 10 Gy) [17]. Minimizing the radiation dose to the least possible level is of high clinical relevance since increased radiation dose to D_100%_ and maximum dose of the hippocampus corresponded to greater decline in memory function [16, 18]. It leads to the hypothesis that hippocampal sparing in patients receiving WBRT might delay or reduce the onset, and/or severity of neurocognitive deficit.

Radiation Therapy Oncology Group (RTOG) 0933 is a single-arm phase II clinical trial that studies the effectiveness of hippocampal sparing in WBRT and has demonstrated promising results in terms of memory preservation using the dose criteria (Table 1) in the protocol [16]. In the meantime, dosimetric characteristics of dual-arc conventional volumetric modulated arc therapy (dac-VMAT) in WBRT with hippocampal sparing have been reported in previous studies following RTOG 0933 criteria [19–21]. The large irradiation field of dac-VMAT for whole brain planned target volume (PTV) required a wide jaw opening which may result in suboptimal multi-leaf collimator (MLC) movements as described in previous publication [22]: (1) Hardware restrictions for MLC movements; (2) Restricted MLC velocity from one gantry angle to another; (3) MLC may reach their limit of travelling distance when they are attempting to move to the distal part of the PTV. In extreme case, the MLC may not be able to shield the desire organs-at-risk (OARs) in distal part of PTV.

**Table 1:** Dose criteria of RTOG 0933 protocol. Dose prescription of 30 Gy in 10 fractions

RTOG 0933 protocol	Per protocol	Acceptable variation	Unacceptable deviation
Whole brain PTV	D_2%_ < 37.5 Gy	D_2%_ = 37.5 Gy	D_2%_ > 40 Gy
	D_98%_ > 25 Gy	D_98%_ < 25 Gy	V_30_ < 90%
Hippocampus	D_100%_ < 9 Gy	D_100%_ = 10 Gy	D_100%_ > 10 Gy
	D_max_ < 16 Gy	D_max_ = 17 Gy	D_max_ > 17 Gy
Optic nerves and optic chiasm	D_max_ < 37.5 Gy	D_max_ = 37.5 Gy	D_max_ > 37.5 Gy

In order to prevent suboptimal MLC movements during hippocampal sparing, Shen et al. [23] has employed the partial-field technique in volumetric modulated arc therapy (VMAT) for WBRT and has reported reduced hippocampal dose; however, exact doses to the other OARs have not been described. In fact, radiation-induced toxicity to the other OARs, including the eyes, during WBRT have been described in previous publications with negative impact on patients’ quality of life [24, 25]. Therefore, radiation dose to the other adjacent OARs should not be overlooked and should also be considered during treatment planning of WBRT with hippocampal sparing.

Until recently, several researchers have employed both split-arc and partial-field technique together to eliminate scatter radiation and MLC limitations in VMAT planning. This technique is beneficial in sparing adjacent OARs in breast cancer [26], cervical [27, 28], anal [28, 29], and vaginal cancer [28]. To the best of the author’s knowledge, the formal literature is devoid of any reference to the application of both split-arc and partial-field technique in VMAT (sapf-VMAT) for WBRT with hippocampal sparing. In the present study, the dosimetric effect of the sapf-VMAT is studied to verify its sparing ability to hippocampus as well as to other OARs on WBRT.

The objective of the present study is to compare the dose sparing capability of dac-VMAT and sapf-VMAT at hippocampus during WBRT.

## Methodology

### Patient selection and computed tomography simulation

Twenty patients, who had been previously treated with WBRT in 2012–2019, were randomly selected and enrolled in the present study. All patients had a previous primary cancer diagnosis that had metastasized and infiltrated the brain. Written consent was obtained from each patient for the present study.

All patients were simulated in the supine position. TIMO Head & Neck Support Cushions (Med-Tec, Orange City, IA) and thermoplastic mask (Klarity Medical & Equipment Co. Ltd., Guangzhou, China) were used for immobilization. The computed tomography (CT) simulation images (native, 120 kV, 80 mA, slice thickness 3 mm, in-plane resolution 1 mm) were acquired using dual-source CT scanner (SOMATOM Definition, Siemens Healthcare, Forchheim, Germany). CT simulation images were stored as Digital Imaging and Communications in Medicine images and were electronically transferred to the Eclipse™ (Varian Medical System, Palo Alto, CA) version 15.5 treatment planning system for WBRT planning.

### Target delineation

The selected patients’ treatment plans were retrieved and re-planned for this retrospective planning study. CT simulation images of each patient were co-registered with the most recent T1-weighted cranial magnetic resonance (MR) images (contrast medium-enhanced base, slice thickness 3 mm, in-plane resolution 0.8 mm) with reference to the bony anatomy. The eyes, lenses, optic nerves, optic chiasm, brainstem and hippocampus were defined as OARs. The hippocampus was manually delineated by single radiation oncologist strictly following the RTOG 0933 atlas definition (available at: http://www.rtog.org). A hippocampal Planning Risk Volume (PRV) was defined as the hippocampus plus uniform 5 mm margin using inbuilt margin expansion function [16]. The whole brain PTV for optimization was created by delineating the whole brain and excluding the hippocampal PRV.

### Dose prescription

The treatment prescription to the whole brain PTV was set to deliver 30 Gy over the course of 10 fractions. All VMAT plans were normalized to ensure that 97% of the whole brain PTV was covered by 95% of the prescribed dose. The acceptable compliance criteria for whole brain PTV and OARs planning doses were listed in Table 1 following RTOG 0933 protocol.

### Treatment planning

**Fig. 1:**
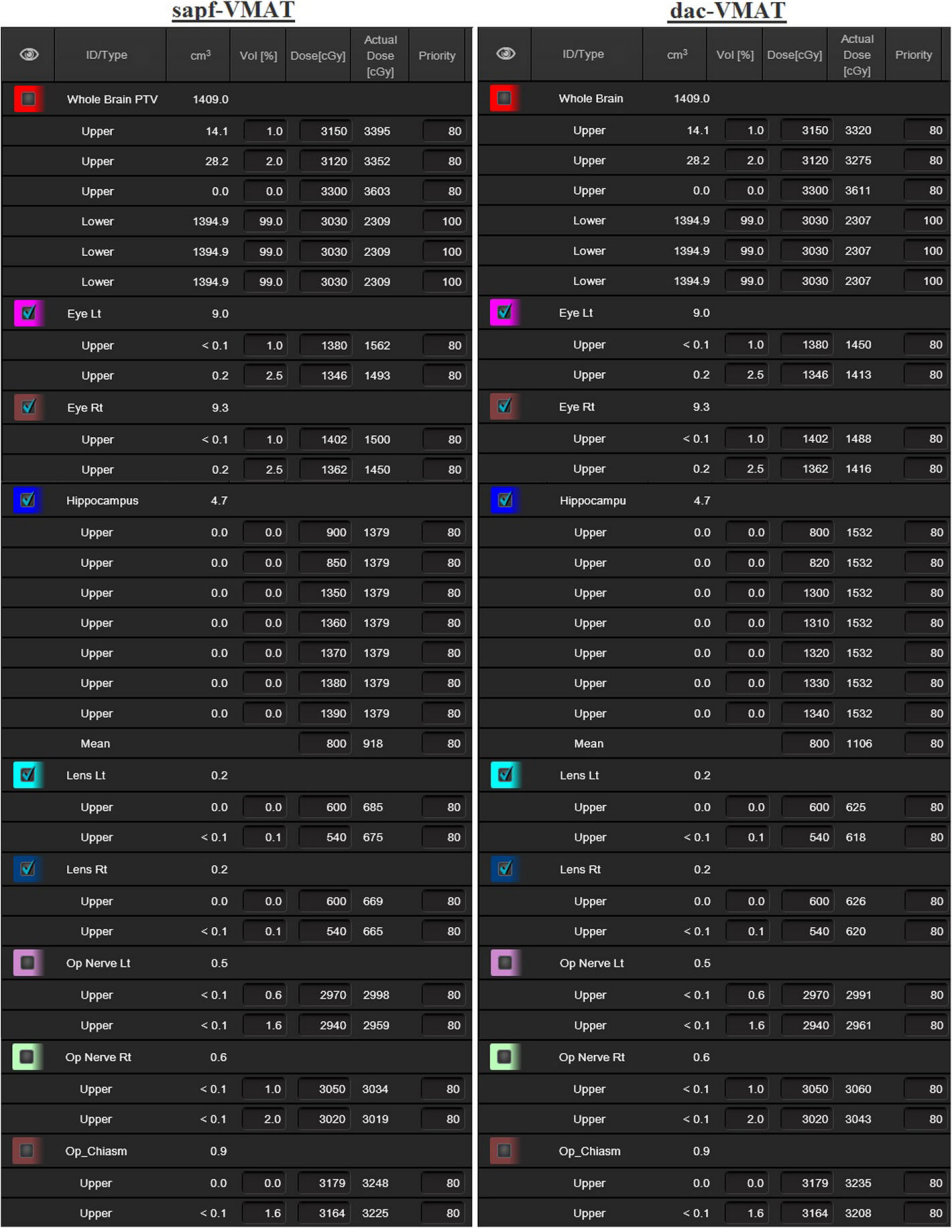
Optimization objectives of major structures for sapf-VMAT (left) and dac-VMAT (right)

All VMAT plans (RapidArc™, Varian Medical System, Palo Alto, CA) were optimized using Eclipse™ (Varian Medical System, Palo Alto, CA) version 15.5 treatment planning system. A total of 40 treatment plans (20 dac-VMAT plans and 20 sapf-VMAT plans) were produced in the present study. Plans were scheduled using 6-MV photon beams with a maximum dose rate of 600 MU/min on a Varian TrueBeam™ linear accelerator with a Millennium 120-leaf MLC (Varian Medical Systems, Palo Alto, CA). Jaw tracking was enabled. The Photon Optimizer (PO, ver.15.5.11, Varian Medical Systems) was used for VMAT optimization. Optimization objectives of major structures were standardized for each technique and were shown in Fig. 1. To avoid introducing bias, the optimization objectives were not modified or individualized between patients of each technique. For dose calculation, the anisotropic analytic algorithm (AAA, ver.15.5.11, Varian Medical Systems) was used with a dose calculation grid of 1 mm. The planning time was similar for each treatment plan in both techniques.

### Dual-arc conventional VMAT (dac-VMAT)

**Fig. 2:**
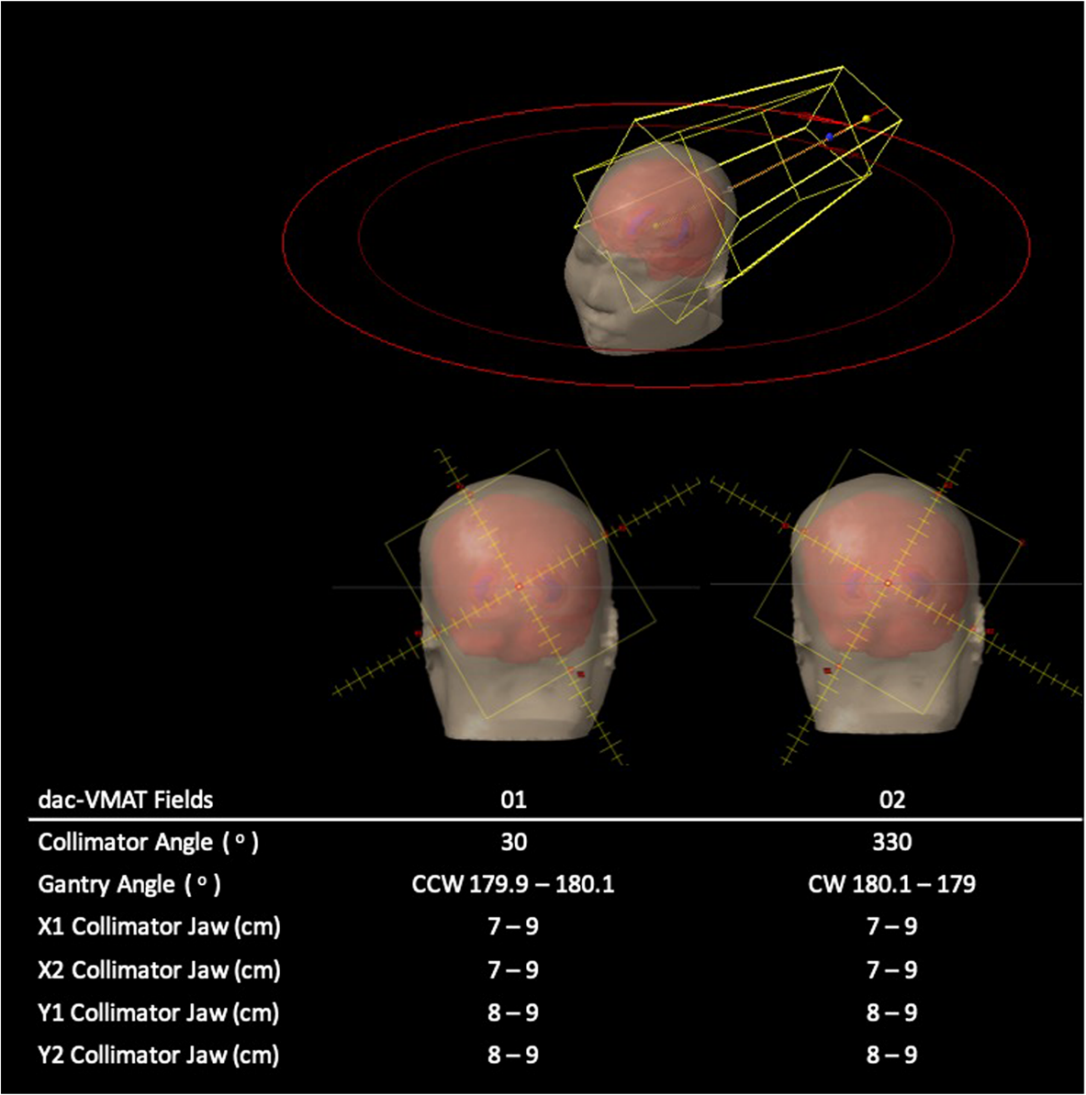
Beam arrangement of dac-VMAT (CCW = counterclockwise; CW = clockwise)

The dac-VMAT plans comprised 2 coplanar arcs of 359.8° each. Collimator rotation of 30° and 330° were used with reference to previous studies [19–21]. The isocentre was placed at the center point equidistant from both hippocampi. The maximum dose rate for the arcs was set to 600 MU/min. Field size was opened up so that the whole brain PTV was completely covered (Fig. 2).

### Split-arc partial-field VMAT (sapf-VMAT)

**Fig. 3:**
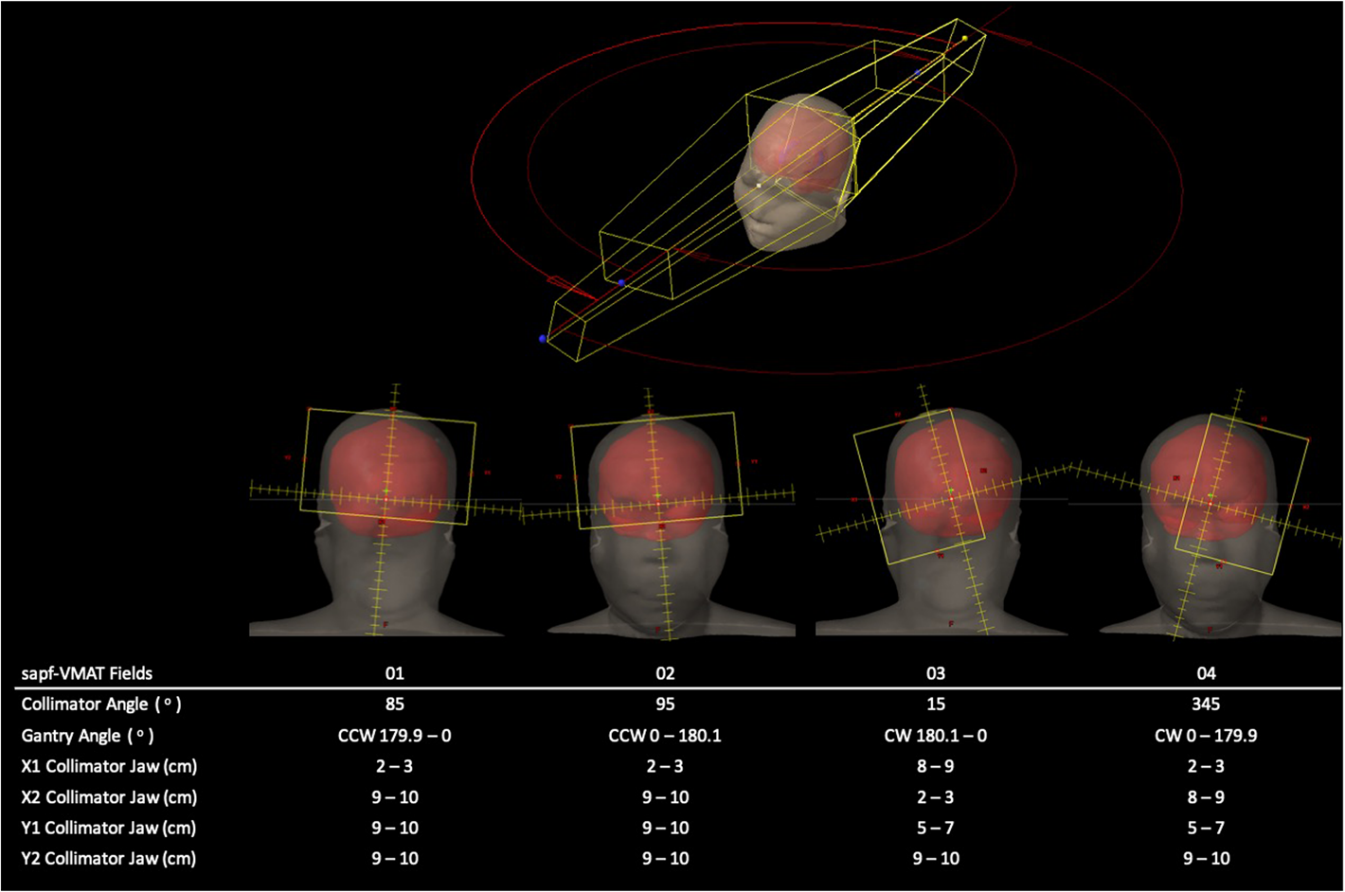
Beam arrangement of sapf-VMAT (CCW = counterclockwise; CW = clockwise)

Four arcs of 179.9° each were used with the same isocentre as the dac-VMAT plans. The maximum dose rate for the arcs was set to 600 MU/min. Collimator angles were chosen to facilitate better use of the MLC. In the present study, collimator angle of 85°, 95°, 15° and 345° were chosen for field 01, 02, 03 and 04 respectively. Field size of each beam arc was reduced so as to allow the MLC to block the centrally located hippocampus without sacrificing the whole brain PTV coverage (Fig. 3):

1. Field 01 and field 02: Due to the larger volume of the superior part of whole brain PTV, two field arcs (359.8°) were used to deliver radiation dose. The length of X1 collimator was reduced to 2 – 3 cm, so that the hippocampus was included. X2 collimator was opened up so that the rest of the superior part of whole brain PTV was covered.
2. Field 03: The field aimed to deliver radiation dose to the right hemisphere of whole brain PTV. The length of X2 collimator was reduced to 2 – 3 cm, while the X1 collimator was opened up, so that the entire right hemisphere of whole brain PTV and the right hippocampi were included. Rotational asymmetry of field 03 was compensated by field 04.
3. Field 04: The field aimed to deliver radiation dose to the left hemisphere of whole brain PTV. The length of X1 collimator was reduced to 2 – 3 cm, while the X2 collimator was opened up, so that the entire left hemisphere of whole brain PTV and the left hippocampi were included.

### Treatment planning evaluation and quality assurance

With reference to the RTOG 0933 protocol criteria, dosimetric parameters of the both VMAT techniques were extracted and compared. The volume of whole brain PTV receiving 30 Gy (V_30Gy_) was recorded for each plan. Dose homogeneity was quantified in terms of homogeneity index (HI), which was defined in the International Commission on Radiation Units and Measurements Report 83 [30] as follows (Eq. 1).

1 $$ HI=\frac{\left({D}_{2\%}-{D}_{98\%}\right)}{D_{50\%}} $$

HI values close to 0 indicated superior homogeneity. Therefore, it was recommended to minimize HI values so as to correspond to more homogeneous dose distribution across the whole brain PTV.

In the present study, the dosimetric parameters of OARs were extracted for comparison including minimum, maximum and mean (D_mean_) doses to the hippocampus; the maximum and mean doses to the eyes, and maximum doses to the optic nerves, optic chiasm, and lenses. Total monitor unit (MU) of each plan was collected and compared between both VMAT techniques. Quality assurance (QA) of treatment plans were performed by dose calculation verification system – MobiusCalc version 2.1 (Mobius Medical Systems, LP, Houston, TX). Treatment plans were exported to MobiusCalc and re-calculated in the patient CT using independently verified beam models and a Graphical Processing Units (GPU)-accelerated collapsed-cone dose algorithm. Target dose, DVH limits, 3D gamma, and deliverability of all treatment plans were verified. All treatment plans were required to have a gamma value > 95% with tolerance for distance to agreement as 3 mm and dose difference as 3%.

Long treatment delivery time has been associated with increased intrafraction motion [31]. In order to investigate both techniques in this respect, beam-on time and delivery time of a single fraction of WBRT with hippocampal sparing were recorded for dac-VMAT and sapf-VMAT. The beam-on time was defined as the summation of the time elapsed from each treatment field beam-on to its beam-off. The delivery time was defined as the time elapsed from the moment of first treatment field beam-on to the end of the last treatment field beam-off, including the time that the gantry travels to the designated starting point. Both beam-on time and delivery time did not include pre-treatment patient setup and daily imaging procedures. Beam-on time and delivery time were measured during QA delivery of the treatment plans.

### Statistical analyses

Statistical comparison between treatment plans of both VMAT techniques were performed using paired t-test. All statistical analyses were performed using SPSS Version 25 statistical software (IBM, USA). *p*-values of < 0.05 were considered to be statistically significant.

## Results

**Fig. 4:**
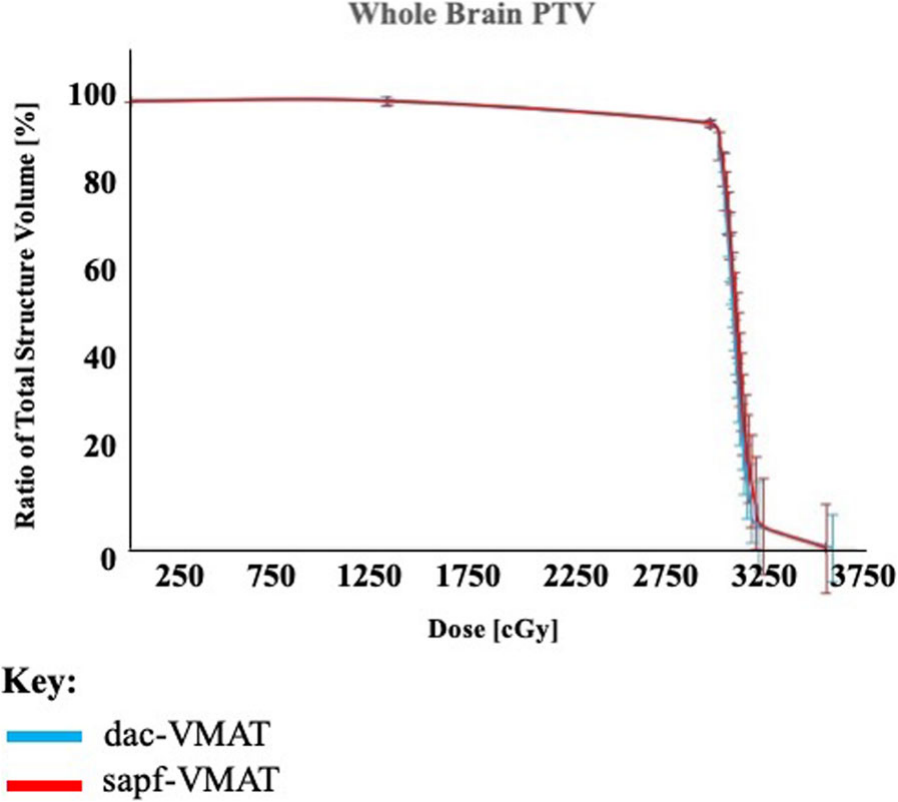
Mean dose volume histogram of whole brain PTV: dac-VMAT (cyan) compared to sapf-VMAT (red). Error bars indicate the standard error

**Fig. 5:**
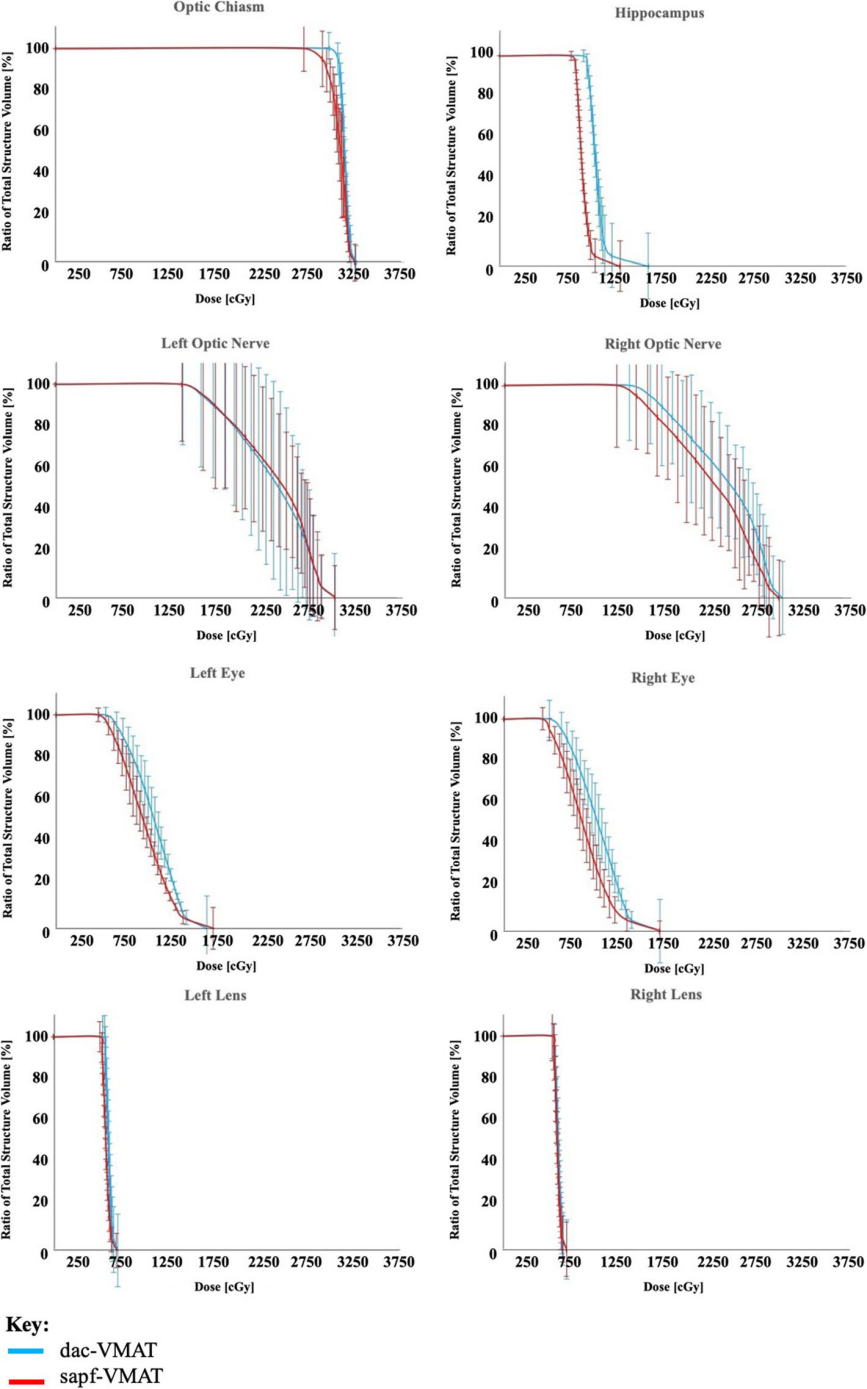
Mean dose volume histograms of organs-at-risk: dac-VMAT (cyan) compared to sapf-VMAT (red). Error bars indicate the standard error

The QA of all treatment plans showed good correlation and reached a passing rate of 95% between treatment planning system-calculated dose and QA system calculated dose (Distance to agreement < 3 mm and dose difference < 3%). Results of dosimetric analysis of whole brain PTV and OARs for the 20 patients in the present study were summarized as mean ± standard deviation (SD) (Table 2). The mean dose-volume histograms (DVH) of the whole brain PTV (Fig. 4) and OARs (Fig. 5) using dac-VMAT and sapf-VMAT were compared.

**Table 2:** Averaged results and comparison of dosimetric parameters using dac-VMAT and sapf-VMAT. Each value was calculated based on the data from 20 patients and was expressed as mean ± standard deviation (SD)

Structures	Dosimetric Parameters	dac-VMAT	sapf-VMAT	*p*-value
Whole Brain PTV	V_30Gy_ (%)	94.67 ± 0.25	94.79 ± 0.13	*p* = 0.358
	D_2%_ (Gy)	33.04 ± 0.32	33.12 ± 0.34	*p* = 0.842
	D_98%_ (Gy)	26.11 ± 0.06	25.84 ± 0.03	*p* = 0.401
	HI	0.22 ± 0.02	0.23 ± 0.01	*p* = 0.435
Hippocampus	D_100%_ (Gy)	9.23 ± 0.13 **	7.86 ± 0.08 **	*p* < 0.001
	D_max_ (Gy)	16.33 ± 0.63 **	13.23 ± 0.46 **	*p* < 0.001
	D_mean_ (Gy)	10.85 ± 0.21 **	9.16 ± 0.10 **	*p* < 0.001
Left Optic Nerve	D_max_ (Gy)	30.80 ± 0.66	31.23 ± 0.48	*p* = 0.791
Right Optic Nerve	D_max_ (Gy)	30.82 ± 0.55	30.51 ± 0.61	*p* = 0.567
Optic Chiasm	D_max_ (Gy)	32.36 ± 0.26	32.48 ± 0.25	*p* = 0.939
Left Eye	D_max_ (Gy)	16.83 ± 0.75	17.12 ± 0.47	*p* = 0.481
	D_mean_ (Gy)	10.46 ± 0.56 *	9.34 ± 0.32 *	*p* = 0.026
Right Eye	D_max_ (Gy)	17.26 ± 0.63	17.21 ± 0.26	*p* = 0.991
	D_mean_ (Gy)	10.36 ± 0.46 *	9.07 ± 0.33 *	*p* = 0.042
Left Lens	D_max_ (Gy)	7.32 ± 0.54	7.33 ± 0.25	*p* = 0.679
Right Lens	D_max_ (Gy)	7.53 ± 0.44	7.12 ± 0.41	*p* = 0.985
Total MU		919.69 ± 130.95	1085.58 ± 153.57	*p* = 0.053
Beam-on time (minute)		3.14 ± 0.11	3.04 ± 0.22	*p* = 0.446
Delivery time (minute)		3.41 ± 0.11	3.62 ± 0.22	*p* = 0.437

* *p* < 0.05;

** *p* < 0.005 (paired t-test)

### Target coverage and dose homogeneity

**Fig. 6:**
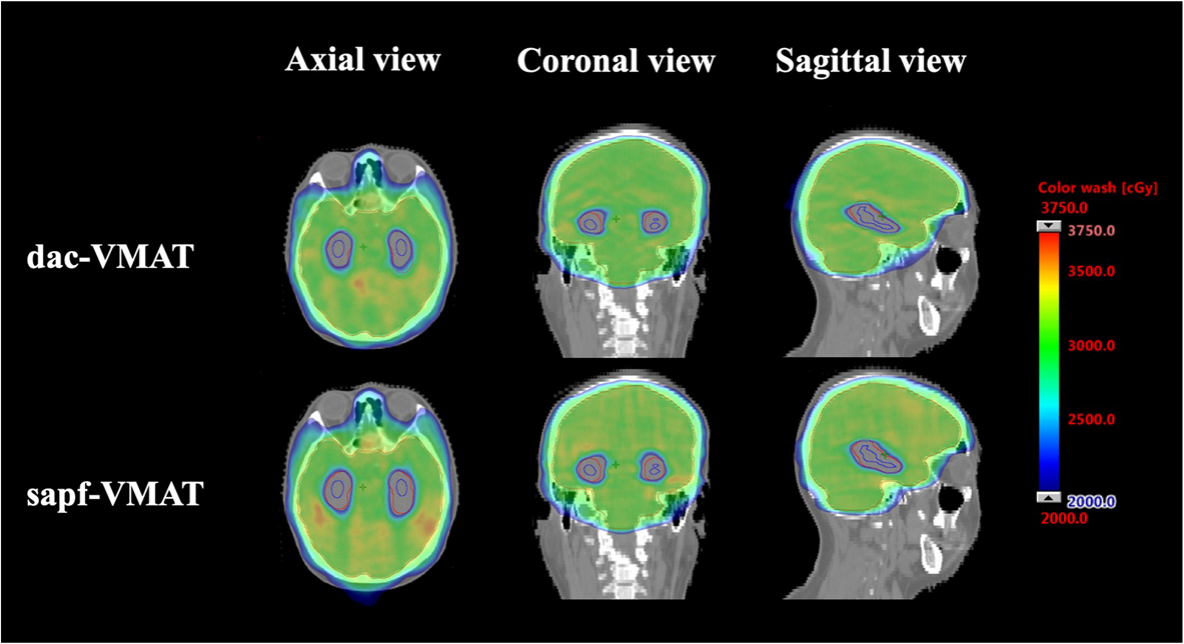
Dose color wash diagrams of dac-VMAT and sapf-VMAT in axial (left), coronal (middle), and sagittal (right) view

The typical dose distribution color washes from 20 Gy to 37.5 Gy of both treatment techniques were demonstrated in Fig. 6. In the present study, all treatment plans had maximum dose less than 37.5 Gy as per RTOG 0933 protocol. All treatment plans were capable to produce adequate target coverage. In terms of the whole brain PTV coverage across the 2 treatment techniques, sapf-VMAT provided average V_30Gy_ of 94.79%, which was comparable to dac-VMAT (94.67%). There were no significant differences (*p* > 0.05) between sapf-VMAT vs. dac-VMAT in V_30Gy_. sapf-VMAT had a mean HI of 0.23, compared to 0.22 for dac-VMAT. No significant differences (*p* > 0.05) were found between both techniques. These findings indicated that both treatment techniques in the present study have similar effectiveness in achieving target coverage and dose homogeneity.

### Hippocampus

sapf-VMAT (7.86Gy, *p* < 0.001) had a significantly lower average D_100%_ compared to dac-VMAT (9.23 Gy). A decrease in hippocampus D_max_ using sapf-VMAT (13.23 Gy, *p* < 0.001) was statistically significant when compared to dac-VMAT (16.33 Gy). The resulting mean dose to the hippocampus were 9.16 Gy for the sapf-VMAT. The mean dose of sapf-VMAT was significantly lower than dac-VMAT (10.85 Gy, *p* < 0.001).

### Optic chiasm, optic nerves, eyes and lenses

The average maximum doses to optic chiasm in dac-VMAT and sapf-VMAT were 32.36 Gy and 32.48 Gy respectively. No significant differences in optic chiasm D_max_ were found between both techniques in the present study (*p* > 0.05). In terms of the averaged maximum doses for both optic nerves, sapf-VMAT were comparable to dac-VMAT (*p* > 0.05). In both eyes, sapf-VMAT demonstrated significantly lower D_mean_ compared to dac-VMAT (*p* < 0.05). No significant differences in D_max_ of both eyes and lenses were found between dac-VMAT and sapf-VMAT (*p* > 0.05).

### Total monitor unit, beam on time and delivery time

The average total MU in dac-VMAT and sapf-VMAT were 919.69 and 1085.58 respectively. The averaged beam-on time were 3.14 min and 3.04 min for dac-VMAT and sapf-VMAT respectively, while the averaged treatment delivery time were 3.41 min and 3.62 min respectively. No significant differences (*p* > 0.05) were found between both techniques for beam-on time and delivery time.

## Discussion

In this planning study, two different techniques (dac-VMAT and sapf-VMAT) were compared in the treatment of 20 patients with brain metastases. All treatment plans were able to achieve the acceptable range of RTOG 0933 (Table 1). Radiation dose to the hippocampus and other OARs were reduced while the whole brain PTV coverage was not compromised.

The present study has suggested a radiation therapy technique – sapf-VMAT, which has consistently demonstrated lower hippocampus dose compared to dac-VMAT plans, with an average reduction of around 14.84, 18.98 and 15.58% in D_100%_, D_max_ and D_mean_ of hippocampus respectively. Meanwhile, hippocampus D_100%_ and D_max_ have been reduced to an average of 7.86 Gy and 13.23 Gy in sapf-VMAT, which are less than the cutoff value of radiation induced neurocognitive deficit onset as described by Gondi et al. [16].

**Fig. 7:**
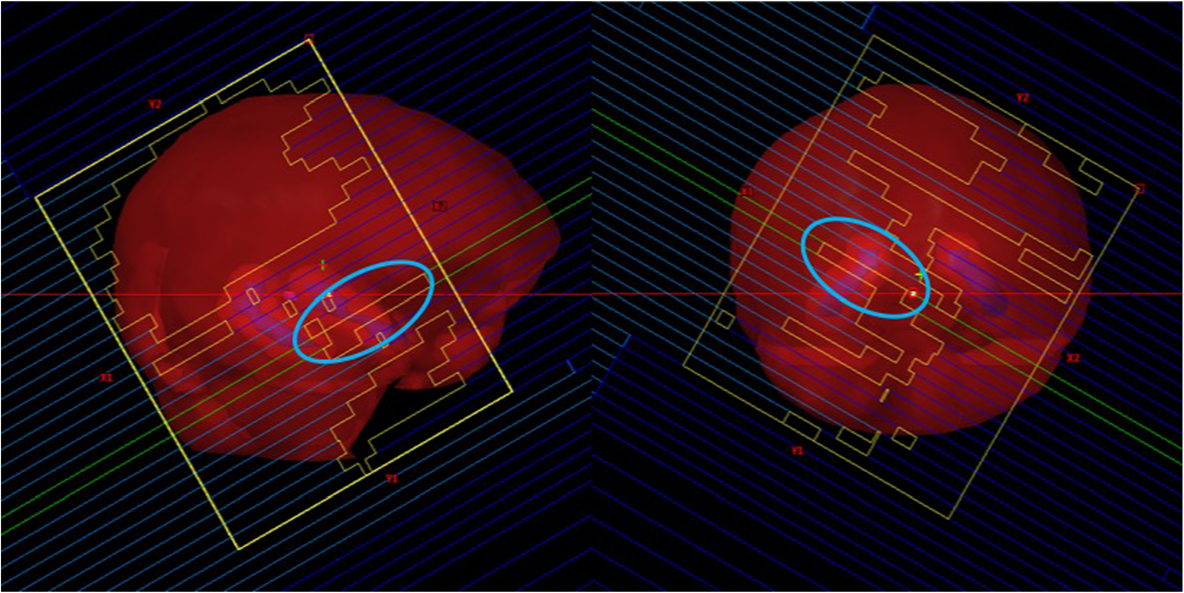
The island blocking problem exists in dac-VMAT that resulting in an area of hippocampus that is not blocked by the MLC (cyan colour circle)

In the present study, dac-VMAT technique comprises 2 coplanar full arcs with large field size covering the whole brain with reference to previous published studies [19–21]. The large irradiation field of whole brain PTV requires a larger jaw opening. This technique may produce a substantial low dose volume in the hippocampus, as a consequence of multi-leaf collimator (MLC) leakage and scatter radiation. The limitation of MLC movement in large field size dac-VMAT may also induce the island blocking problem [32, 33] (Fig. 7). The island blocking problem exists when ≥2 areas of whole brain PTV share the same MLC leaf pair, resulting in an area of hippocampus that is not blocked by the MLC, and hence increased low dose spillage to the hippocampus. Since reduced field size in either X1 and X2 collimator jaw has been employed for sapf-VMAT plans, an independent jaw can be moved to block off part of the field to reduce scatter radiation. This feature is useful for adjacent normal healthy tissue sparing, that is, the hippocampus. In addition, the reduced field size can shorten travelling distance of MLC, and therefore MLC movement is less likely to be restricted by its velocity and physical limitation. Thus, MLC in sapf-VMAT are capable of shielding the hippocampus in all gantry angles, while remaining enough dose coverage to whole brain PTV.

In the coplanar VMAT planning, constraint of radiation dose to the eyes and hippocampus is sometimes considered to be a difficult goal. Since the eyes and the hippocampus are collated on the same plane, which creates difficulties during optimization using dac-VMAT. In sapf-VMAT plans, the proposed split-arc design can help the optimizer to avoid irradiation of whole brain PTV with the expense of hippocampus and both eyes by collimator rotation between the arcs. The reduced treatment field size can also reduce the swept angle that both eyes lie within the treatment field, resulting in dose reduction in hippocampus and both eyes using sapf-VMAT.

In addition to tissue sparing, another major advantage of using sapf-VMAT is that the overall swept angle remains equal to the dac-VMAT for WBRT (i.e. 719.6°), although the number of treatment arcs in sapf-VMAT is increased to 4. Hence, no major increment in treatment delivery time (13 s more than dac-VMAT on average) is induced using sapf-VMAT. This technique will not impact on patient comfort on the treatment couch and affect the reproducibility of treatment position.

The sapf-VMAT plans generated in this study has resulted in higher averaged MU usage than the dac-VMAT plans (averaged difference of 166 MU). It is believed that the higher MU usage resulting from sapf-VMAT plans is a consequence of the highly conformal dose distributions and superior OAR sparing. Admittedly, higher MU has its drawbacks such as the potential increase in total body dose because of scattering and leakage from MLC. Therefore, in future improvement of the sapf-VMAT, efforts should be spent on reducing the MU usage while maintaining the plan quality.

## Conclusion

The present study has proposed a radiation therapy technique, namely sapf-VMAT, that has employed split-arc and reduced field size. This technique has demonstrated significant dose reduction to the hippocampus and eyes compared to dac-VMAT. Therefore, the clinical usability and functional outcome of this strategy should be further investigated in sapf-VMAT.

## Data Availability

The data that support the findings of this study are available from the Oncology Centre, St. Teresa’s Hospital (HKSAR) but restrictions apply to the availability of these data, which were used under permission for the current study, and so are not publicly available. Data are however available from the authors upon reasonable request and with permission of the Oncology Centre, St. Teresa’s Hospital (HKSAR) at the following e-mail address: sthochk@gmail.com.
